# Reductions in brainstem volume as a key macrostructural indicator in at-risk populations for Alzheimer’s disease

**DOI:** 10.1186/s13195-025-01829-0

**Published:** 2025-07-26

**Authors:** Thomas M Lancaster, Kevin Murphy, Hannah Chandler

**Affiliations:** 1 Cardiff University Brain Research Imaging Centre (CUBRIC), School of Physics and Astronomy, Cardiff University, Maindy Road, Cathays, Cardiff, CF24 4HQ UK; 2 Department of Psychology, University of Bath, Claverton Down, Bath, BA2 7AY UK

**Keywords:** Alzheimer’s disease, Mild cognitive impairment, Polygenic risk score, Brainstem, Preclinical, MRI

## Abstract

**Background:**

Alterations to brain macrostructure, assessed via T1-weighted magnetic resonance imaging are observed in preclinical models of Alzheimer’s disease (AD), reflecting susceptibility, prodromal stages of AD or correlates of early AD pathophysiology. While changes in cingulate and medial temporal lobe structures may be functionally implicated in cognitive decline, little is known about the viability of brain-based biomarkers that support autonomic functions implicated in preclinical AD risk such as the brainstem.

**Methods:**

In a series of multiple linear regressions, we assess the volume of the brainstem in two asymptomatic at-AD-risk samples, assessed via the presence of either mild cognitive impairment (MCI, *N* = 148), or extremely high polygenic risk (*N* = 13) with matched demographics (mean age = 67 [range 58–76], in both cases). We further determine the strength of the association, compared to 150 other structural MRI features.

**Results:**

We observed brainstem volume reductions (MCI: b = -0.29, *P* = 0.018; Genetic risk: b = -1.29, *P* = 0.002) in both samples. The magnitude of each preclinical AD marker (MCI / AD-polygenic risk)– brainstem association was empirically larger (Z > 2.3, *P* < 0.05, in both cases) than 150 frequently segmented MRI features. We further replicate the negative AD-polygenic risk score– brainstem association in UK Biobank (*N* = 31968; b = -0.002, *P* = 0.03), with weaker evidence that the association was larger than all other MRI features (Z = 1.622; *P* = 0.052).

**Conclusions:**

These observations suggest that AD risk, assessed via the presence of MCI or extremely high AD-polygenic risk score is linked to reduced brainstem volume before most typically observed morphological brain alterations. This conforms with evidence implicating the brainstem as one of the earliest sites of morphological neurodegeneration and provides a plausible biological mechanism linking prodromal autonomic symptoms to AD risk in later life. These observations warrant future investigation into the molecular correlates of AD-linked brainstem dysfunction, assessment as a candidate biomarker, and the exploration of brainstem mediated treatment strategies in AD prevention.

**Supplementary Information:**

The online version contains supplementary material available at 10.1186/s13195-025-01829-0.

## Introduction

Macrostructural volumetric reductions observed via magnetic resonance imaging (MRI) reflect neurodegeneration as a key feature within the Amyloid-Tau-Neurodegeneration (ATN) framework of Alzheimer’s disease (AD) pathogenesis [1, 2]. This ubiquitous term broadly encompasses subcortical volumetric, cortical thickness and surface area reductions, frequently in temporal-limbic brain regions, linked to a broad range of memory and executive deficits [3, 4]. Reductions in the morphometry of these networks are typically observed in groups of individuals with an AD diagnosis, in at-risk individuals’ such as those with mild cognitive impairment (MCI) or those with increased genetic risk, via possession of *APOE* ε4 or a higher AD polygenic score (AD-PRS) [5]. Alterations in brain macrostructure are therefore considered as part of the prodromal aetiology for AD and occur before the onset of cognitive disruptions. While alterations in high-order cognition such as memory, executive function, and language (and their neural correlates) are increasingly recognised as early markers of AD, there are numerous physical presentations that are antecedent to AD. For example, alterations to sleep architecture, hearing, and vision health are established risk factors that occur in midlife [6–11]. These autonomic functions are controlled in part, by the brainstem, responsible for regulating essential functions such as arousal / attention, sleep-wake cycles, and autonomic control [12, 13]. Many of these functions are genetically linked to AD, with an overlapping genetic architecture between AD and brain-stem-linked functions such as blood-pressure / hypertension [14, 15], sleep architecture [16, 17], hearing-loss [18, 19], pupillary reflexes [20]. There is also consensus that the brainstem volume is reduced in AD and in at-risk groups such as individuals with MCI [21–25], with large meta-analysis (*N* > 27,000) demonstrating brainstem volume reductions are consistently and generally observed in AD [26] and at risk groups such as patients with mild cognitive impairment (MCI), also exhibit significant reductions in brainstem volume. These volumetric changes are also associated with poorer performance in cognitive tasks, suggesting that brainstem atrophy may also contribute to the cognitive deficits observed in the prodromal stages of AD [23]. This brainstem atrophy correlates with the early deposition of tau (T), a pathological hallmark of AD, indicating that the brainstem is one of the first regions affected by AD pathogenesis. For instance, the locus coeruleus, responsible for noradrenaline production, is one of the earliest sites of AD-linked tau deposition [27]. Brainstem nuclei such as the locus coeruleus are among the first foci to develop tau pathology, leading to subcortical then cortical regions [27], potentially starting in early adulthood [28]. Genome-wide association studies (GWAS) further indicate that individual variation in brainstem volumes and risk for AD have genetic overlap [29] suggesting a common molecular aetiology.

With converging evidence demonstrating a number of well-established common genetic variants contribute to risk for Alzheimer’s disease (AD), which have impact *en masse* comparable to the established *APOE* ε4 allele risk factor [30], an individuals’ genetic risk score (polygenic risk score; PRS) provides a stable indicator of risk across the lifespan, useful for informing preclinical models, antecedent to symptoms. There is also evidence suggesting that individuals with higher AD-PRS have elevated Tau biomarkers, especially in the presence of elevated amyloid burden [31–33]. While Braakian staging based on the progression of post-mortem based histopathological features [27] corresponds with the progression of T1-weighted MRI measures of grey matter loss [34], the brainstem remains largely unconsidered as a node in these models of progressive neurodegeneration [35]. Considering the brainstem’s involvement in (i) regulating AD-linked autonomic functions; (ii) its reduction in AD / MCI and (iii) its link to Tau, volumetric reduction in the brainstem may be a typical feature in prodromal or early AD, and may also occur early in the disease process, at least before AD diagnosis and as a function of common genetic risk. Here, we principally assess brainstem volume in two preclinical samples. The first sample consists of individuals with MCI and normal aging controls of comparable age, matched for several key confounds (such as age, sex, years of education) [36]. The second sample was collected as part of a small, proof-of-concept recall-by-genotype (RbG) study recruiting asymptomatic individuals with high polygenic risk for AD [37]. Together, these samples will help establish if brainstem volume reductions are present in individuals before an AD diagnosis. Moreover, we compare any brainstem alterations to other neo/subcortical alterations that could co-occur in these individuals, to help establish a temporal order to AD-risk linked neurodegeneration.

## METHODS

### Participants A: HCP-Aging

The Aging Human Connectome Project (HCP-Aging) has been extensively described elsewhere. Briefly, the sample consists of participants aged between 36 and 100, inclusively. The broader HCP-Aging sample excluded individuals with any present of historical psychiatric, neurological or neurodegenerative diagnosis. The presence / absence of mild cognitive impairment was assessed via the Montreal Cognitive Assessment (MoCA; [38]), where participants scoring between 19 and 25 were classified with MCI and > 26 as clinically healthy. Participants were not considered to have a diagnosis at enrolment [36, 39]. One participant who scored 31 (full marks with low IQ) was excluded. To ensure the sample was comparable to our follow-up PROTECT-RbG sample (see below - Participants B), we restricted the HCP-Aging imaging sample (from the broader HCP-Aging dataset) to individuals with the same range observed in the PROTECT-RbG sample (mean age 67, range 58–76, see below– Participants B). We further used propensity score matching (PSM; via MatchIt [40]) to ensure that the sample was adequately controlled for confounding in demographic factors such as age, sex and education status. After PSM matching, with a 1:1 ratio, the sample consisted of 74 healthy control individuals and 74 individuals who met the MoCa criteria for MCI (MoCA score range 19–25), with comparable frequencies of sex, and mean years of age / education (Table 1).

**Table 1:** *Education measures as years in HCP-Aging and highest level of qualification in Protect-RbG. MoCA = Montreal cognitive assessment

	HCP-Aging		PROTECT-RbG	
	Healthy Controls (*N* = 74)	Mild Cognitive Impairment (*N* = 74)	High AD-PRS (*N* = 5)	Low AD-PRS (*N* = 8)
**Sex**				
F	36 (48.6%)	35 (47.3%)	4 (80.0%)	6 (75.0%)
M	38 (51.4%)	39 (52.7%)	1 (20.0%)	2 (25.0%)
**Age at scan**				
Mean (SD)	67.6 (4.78)	67.2 (5.72)	62.8 (6.10)	67.9 (5.99)
Median [Min, Max]	68.0 [58.1, 76.0]	67.2 [58.2, 76.0]	62.0 [56.0, 70.0]	68.5 [58.0, 74.0]
Education*				
Mean (SD)	17.5 (2.21)	17.4 (1.84)	4.80 (2.17)	5.75 (1.58)
Median [Min, Max]	18.0 [10.0, 21.0]	18.0 [13.0, 21.0]	6.00 [2.00, 7.00]	6.00 [2.00, 7.00]
**MoCA**				
Mean (SD)	27.7 (1.39)	23.8 (1.28)	NA (NA)	NA (NA)
Median [Min, Max]	28.0 [26.0, 30.0]	24.0 [21.0, 25.0]	NA [NA, NA]	NA [NA, NA]
Missing	0 (0%)	0 (0%)	5 (100%)	8 (100%)

### Participants B: PROTECT-RbG

An extensive description of the study design / sample characteristics has been recently published elsewhere [37]. Briefly, we performed neuroimaging assessment on sixteen individuals aged 56–74, who had either an Alzheimer’s disease polygenic risk score (AD-PRS) two standard deviations lower (*N* = 10) or higher (*N* = 6) than the average of a large population (*N* = 4504), providing adequate power to determine group differences in future AD diagnosis. For each participant, AD-PRS was calculated by generating a weighted sum of the number of AD risk alleles they possessed divided by the number of genetic variants considered, using established parameters [41]. This study received ethical approval from Cardiff University’s School of Psychology. Exclusion criteria included: >80 years old, having a history of psychiatric diagnoses, substance abuse, neurological disorders, or head injuries, using chemotherapy or immunomodulatory drugs, genetic disorders, type I/II diabetes, and cardiac, vascular, or pulmonary conditions, including a history of high blood pressure or asthma. To ensure our inferences remained unconfounded by *APOE* status, we restricted our RbG sample to exclusively *APOE* ε3ε3 homozygotes (final sample; *N* = 8 / 5). The low / high AD-PRS groups were similar in terms of age, sex, and educational attainment measured by the highest UK qualification levels (see Table 2 for further details).

**Table 2:** Samples were acquired on 3T Siemens Prisma systems, using an MPRAGE (magnetisation-prepared rapid acquisition with gradient echo), sequence. Ψ Multi-echo MPRAGE acquired as previously described [44]. UKBB = UK biobank

Sample	TR(ms)	TE(ms)	FoV (mm^2^)	Voxel size (mm3)	Slices	FreeSurfer version
HCP-Aging	2500	[1.8, 3.6, 5.4, 7.2] Ψ	256 × 256	0.8 × 0.8 × 0.8	208	6.0.0
PROTECT-RbG	2100	3.24	165 × 203	1 × 1 × 1	197	7.1.1
UKBB	2000	800 (TI)	208 × 256 × 256	1 × 1 × 1	208	6.0.0

### T1-weighted imaging acquisition, pre-processing & analysis

**Fig. 1:**
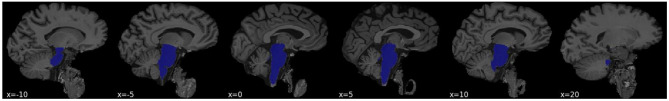
Example segmentation of the brainstem (blue) as estimated in FreeSurfer 7.1.1, across six sagittal slices, from a T1-weighted MPRAGE sequence

For neuroimaging acquisition parameters, see Table 1. T1-weighted MRI scans were pre-processed through freesurfer versions 6.0.0 (HCP-Aging/UKBB) and 7.1.1 (PROTECT-RbG). As well as brainstem volume (mm3)– see Fig. 1, cortical thickness (mm^2^), cortical surface area (mm) of 34 bilateral cortical regions, and the volume (mm^3^) of 7 bilateral subcortical regions were estimated using the processing and reconstruction method outlined by Fischl et al. [42] to segment and label cortical and subcortical volumes based on cytoarchitectural boundaries via the deskian Killiany atlas [43].

### Power analysis

Comparable T1w MRI studies [23] have considered early / mild stages of AD, demonstrating large brainstem volume loss in mild-AD compared to controls (Cohen’s *d* = 0.95). Our HCP-Aging MCI sample had > 99% power to detect a between group difference of this standardised mean difference. effect size. Power analysis for our PROTECT-RbG sample has previously been reported. Briefly, due to the size of the population from which our participants were re-recruited (*N* = 4504), we have > 85% power to detect differences in future AD incidence between our extremely low (-2SD) and high (+ 2SD) AD-PRS groups. However, only including individuals with the *APOE* ε3ε3 genotype reduced this power (67%). We therefore report AD-PRS group differences in brainstem volumes for samples with/without those possessing an *APOE* ε4 allele.

### Statistical analysis

A single, multiple linear regression was performed with age, sex, years of education and total intracranial volume (ICV) as covariates of no interest for both samples. For the HCP-Aging, imaging site was added as a random effect via the lme4 package to adjust for potential site related differences [45]. Freesurfer versions and sequences were consistent within study cohorts. In order to assess the specificity of AD-related brainstem reductions, we further estimated coefficients for *N* = 150 structural segmented grey matter features (34 × 2 cortical thickness-mm; 34 × 2 surface area-mm2 and 7 × 2 subcortical volume– mm^3^), typically considered in large neuroimaging case-control meta-analysis [37, 46, 47], with labels / cytoarchitectural boundaries provided as part of the Desikan-Killany atlas [43]. We repeated each model, replacing our principal dependent variable (brainstem - mm^3^) with each of the 150 T1w features individually, keeping all other model parameters / covariates consistent, apart from global metrics such as total thickness and surface area, which were considered as covariates for all thickness and surface area regional features, respectively. A density plot of absolute effect sizes of all 150 T1w features were created for each sample and the brainstem coefficients for each sample was empirically ranked against its respective density distribution, created a z-score / *p*-value for brainstem - coefficients difference from each coefficient distribution.

### Alzheimer’s disease polygenic risk score analysis in UK Biobank

To replicate potential associations between AD-PRS and brainstem volume, GWAS summary statistics were also acquired based on a recent UK Biobank MRI-GWAS, corrected for demographic, neuroimaging and genetic confounds [38]. We assess AD-PRS– brainstem volume associations using the ‘gtx’ method, equivalent to the “inverse variance weighted” approach in mendelian randomization [39, 40]. However, in a PRS analysis, SNPs do not have to be strongly associated with brainstem volume and pleiotropic effects are allowed [39, 40]. Briefly, ‘gtx’ uses established GWAS summary statistic data for both the exposure (AD) and outcome (GWAS summary data for brainstem volume), which approximates the regression for an exposure (i.e. risk for AD, based on AD GWAS summary statistics) into an AD-PRS, which are weighted by SNP regression coefficients for the brainstem GWAS results. We employed recent AD GWAS summary statistics, which did not overlap with UK Biobank [41]. We consider SNPs at a *P*-value threshold ≤ 0.5, as per our original calculation for the recall-by-genotype AD-PRS calculation [8] and removed SNPs with a minor allele frequency < 1% and imputation quality < 0.9. SNPs within both the major histocompatibility complex (chr 6: 26,000–34,000 kb) and *APOE* regions (chr 19: 44,400–46,500 kb) were also removed from the pruned dataset (r2 = 0.01, kb = 1000). We remove the *APOE* locus due to its pleotropic associations with a wide range of preclinical AD biomarkers [48–50]. We also create a density plot of absolute effect sizes of all T1w features (*N* = 150) from UK Biobank and empirically rank the AD-PRS - brainstem volume association against its respective density distribution, created a z-score / *p*-value.

## Results

**Fig. 2:**
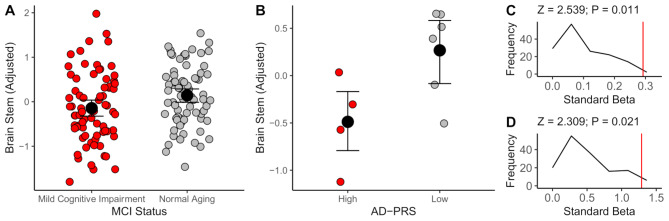
A-B: Normalised residual parameter estimates for brainstem volume, adjusted for age, sex, education and intracranial volume for HCP-Aging and PROTECT-RbG, respectively. Error bars represent 95% bootstrapped confidence intervals. C-D: Distribution plots (y-axis frequency) of coefficients for MCI / AD-PRS (x-axis– magnitude of beta coefficient) in a further *N* = 150 T1w MRI features. Red vertical lines represent the magnitude of association between AD risk and brainstem volume for HCP-Aging and PROTECT-RbG, respectively

Consistent with prior reports of reductions in brainstem volume after the previously observed age inflection of 51.8, in the HCP-Aging sample [51], we observed consistent, albeit small negative associations between brainstem volume and age in both samples (*P* < 0.01, in both cases). Critically, brainstem volume was lower in our HCP-Aging MCI group compared to the HCP-Aging Controls (β = -0.29, *P* = 0.018, Cohen’s *d* = − 0.39 [95% CIs: -0.07, -0.72]) and our PROTECT-RbG high AD-PRS groups (β = -1.29, *P* = 0.002, Cohen’s *d* = -2.00 [95% CIs: -0.46, -3.46]). Including individuals possessing an ε4 allele did not significantly influence this association (β = -1.05, *P* = 0.005). See Table 3 for further regression details and Fig. 2A-B for parameter estimates. We then generated comparable estimates for these contrasts for an extensive range of T1w features (*N* = 150) and compared the brainstem estimates to these 150 coefficients. In both cases, the brainstem coefficient was higher than the majority of T1w cortical/subcortical features (Z _HCP−AGING_ = 2.54, *P* = 0.011; Z _PROTECT−RBG_ = 2.31, *P* = 0.021, see Fig. 2C-D). We further explored which of the 150 T1w MRI features had shared variance with brainstem volume. In the HCP-Aging sample, we observed that subcortical volumes were robustly correlated with brainstem volume, but also limbic and medial temporal lobe structures (see Supplementary Results 1 A). We further observed that the more variance a T1w MRI feature shared with the brainstem, the more impacted it was in AD, based on estimates from an independent sample (ADNI AD vs. HC; [52]; *r* = -0.37, *P* = 0.02– see Supplementary Results 1B), suggesting that the brainstem shares disproportionally more variance with brain regions that experience the most dramatic volume losses in AD.

**Table 3:** Regression coefficients for the association between the brainstem volume (mm^3^) dependent variable and model predictors in (i) HCP-A and (ii) PROTECT RbG samples. AD-risk (and coefficient) denotes the presence of mild cognitive impairment (MCI) or possessing a high AD-PRS. BETA = estimate of coefficient; se = standard error of coefficient; age = age at time of scan; icv = intracranial volume

	MCI			*P*	AD-PRS			*P*
	BETA	SE	t value		BETA	SE	t value	
(Intercept)	3.27	0.81	4.04	0.001	4.99	1.24	4.03	0.007
**AD-risk**	**-0.29**	**0.12**	**-2.40**	**0.018**	**-1.29**	**0.25**	**-5.21**	**0.002**
Age	-0.004	0.001	-3.99	0.001	-0.07	0.02	-3.88	0.008
Sex	0.05	0.15	0.32	0.746	0.43	0.49	0.89	0.409
ICV	0.64	0.08	8.20	0.001	0.39	0.25	1.56	0.17

### Replication of AD-PRS effect in UK Biobank

As we observed a negative association between AD-PRS and brainstem volume in the RbG sample (β = -1.29 ± 0.25, *P* = 0.002), we acquired the summary statistics for a comparable GWAS from UK Biobank (Image Derived Phenotype ID: 0177, aseg_global_volume_Brain-Stem, *N* = 31968). We replicated this observation in this UKBB sample (*N* = 31966; β = -0.002 ± 0.001, P_REPLICATION_ = 0.03). We repeated this analysis, further considering if *APOE* status provided any additional explanatory variance. An AD-PRS model which considered *APOE* was still negatively associated with brainstem volume (β = -0.002 ± 0.001, *P* = 0.016), but this did not provide significantly more explanatory variance (t = -0.202, *P* = 0.84), suggesting a minimal role of *APOE* status in brainstem volume. Compared to estimates for an extended range of T1w cortical/subcortical features (*N* = 150), we observed weaker evidence that the AD-PRS– brainstem association was larger than the majority of all T1w features (Z _UKBB_ = 1.622, *P* = 0.052). While not surviving family wise error correction across the 150 MRI features, we also observed negative associations between AD-PRS and hippocampal volume (β = -0.0019 ± 0.001, *P* = 0.029), as previously reported [46], supporting the validity of our AD-PRS brainstem inference (see Supplementary Results 2 for all associations).

## Discussion

We establish converging evidence that brainstem volume is reduced in groups of mid/older adults at risk for Alzheimer’s disease (AD) compared to those with low/typical AD risk. First, we replicate a previous observation demonstrating that individuals with mild cognitive impairment (MCI) have smaller brainstem volumes on average [23, 29]. Next, we demonstrate that older, asymptomatic individuals with extremely high genetic predisposition for AD, as assessed via a polygenic risk score (AD-PRS), also have brainstem volume reductions. As the PROTECT-RbG sample solely consisted of individuals with an *APOE* ε3ε3 genotype, we do not anticipate this association to be confounded by *APOE* ε4 status, which has numerous effects on grey matter brain macrostructure in later life, although we are not aware of any studies implicating *APOE* ε4 in brainstem macrostructure/volume in aging samples [22, 53] and did not observe any evidence for in our sample. While we cannot rule out confounding from *APOE* status in the HCP-A sample, both AD-PRS associations were independent from any *APOE* locus related influence. The HCP-Aging and PROTECT-RbG samples also benefit from being of comparable age and were internally matched for demographic confounds such as sex and education levels. Last, we demonstrate that the magnitude of the brainstem reductions was mostly larger than any other T1-weighted (T1w) MRI features estimated via common imaging processing tools such as FreeSurfer [42]. Reduced brainstem volume has been linked to a range of psychometric performance assays in attention, processing speed and executive function across the risk AD continuum [22], but broader assessments for the clinical correlates of brainstem volume reductions are warranted.

The brainstem further performs key roles regulating critical homeostatic functions, including sleep-wake cycles, arousal, and autonomic control, which are disrupted in AD. Brainstem atrophy may serve as an early morphological biomarker for AD, since these changes occur before other, established cortical atrophy, they could potentially be used to identify individuals at risk for developing AD. Additionally, the association between brainstem volume and cognitive performance shown by others suggests that preserving brainstem integrity might be a therapeutic target for slowing disease progression. There is evidence that the earliest signs of tau pathology emerge in the brainstem [27, 28] and as AD-PRS has been linked to tau biomarkers [33], we speculate that alterations in brainstem volume may represent a candidate biomarker for individuals with high genetic risk for AD. We speculate that this could reflect susceptibility from non-*APOE* related common AD risk variants. While *APOE* has established, significant impacts on MRI features when considered in an AD-PRS model, AD-PRS excluding the *APOE* locus have been previously linked to other AD biomarker features such as cognition and neurodegeneration [37, 54–56]. Individuals with MCI in the HCP-Aging demonstrate cumulative patterns of AD-specific cortical alterations [57], however, we observed little evidence for specific, significant cortical or subcortical reductions, after controlling for family-wise-error rates in our confound matched sub-sample. This suggests that although an AD-related grey matter profile may be present in the MCI group in this sample, the brainstem may be among one of the structures first impacted. This is supported by our final observation in the HCP-A sample, where T1w MRI features which were most related to brainstem volume were the regions most impacted in AD, as estimated in an independent sample. This suggests that the brainstem shares a disproportionate amount of variance with grey matter in the brain regions most vulnerable in AD. Studies have further linked clinical correlates in *APOE*, MCI and other AD risk groups to white matter microstructure alterations in the brainstem [58] so future work exploring specific diffusion properties and susceptibility-weighted data may provide additional molecular insight into brainstem reduction in early AD risk [59].

We acknowledge our observations in light of the following limitations. First, while we replicate prior observations linking MCI to reduced brainstem volume, our observation between high AD-PRS (excluding the influence of *APOE*) and reduced brainstem volume is the first instance and should be interpreted with caution until replicated in larger, more generalisable samples. Second, each of our samples were carefully demographically matched internally, with similar ages across sample. While this may help optimise study comparability, we cannot generalise these observations to other age groups or clinical stages. Third, we did not have access to genetic information from the HCP-Aging sample, so we cannot rule out that these observations could be (i) explained / confounded by the presence of *APOE* e4 or (ii) linked to non-*APOE* polygenic risk like our PROTECT-RbG study. Fourth, there are also a number of limitations related to the recall-by-genotype study that we have previously acknowledged, included sample size, sources of confounds and unknown prodromal stage [37]. We further acknowledge that without relevant preclinical biomarker assessment, we are unable to comprehensively assess AD-risk in these individuals and AD-PRS can only serve to explain a proportion of AD risk. The AD-PRS used to invite participants was also constructed with a liberal P-threshold. While these AD-PRS typically perform optimally for delineating case-control status [41], the biological pathways that explain this association remain unknown [47]. Fifth, while we observed a comparable negative association between AD-PRS and brainstem volume in UK Biobank, the effect was not as prominent compared to other T1-weighted features assessed, with weaker evidence that it was the strongest anatomically foci linked to AD-PRS. Sixth, due to each cohort’s cross-sectional design and in the absence of other established biomarkers, we note that we are unable to empirically rank / assess the prognostic performance of brainstem volume compared to other preclinical AD biomarkers within the ATN framework. Future work could incorporate brainstem volumes into data-driven, multimodal assessments of future AD risk [60–62]. Last, while we speculate that AD-risk mediated brainstem alterations may explain some of the autonomic dysfunction observed in prodromal AD, future follow up assessment will be required to establish if these alterations precede these disturbances.

In conclusion, we observe reduced brainstem volume in two samples modelling future AD risk, in older participants not currently diagnosed with AD. Brainstem volume reductions may serve as an early biomarker for AD and highlights potential dysfunction in the initiation and progression of AD. We advise that future work should establish neuropathological / clinical correlates of brainstem reduction, which could provide insight into biological processes linking common AD genetic risk and mechanistically explain the genetic correlations / pleiotropy between AD and brainstem governing autonomic functions. These studies could provide insight into drug targets that help preserve brainstem-linked function and reduce future incidents of AD.

## Electronic supplementary material

Below is the link to the electronic supplementary material.

Supplementary Material 1

Supplementary Material 2

## Data Availability

Research reported in this publication was supported by the National Institute On Aging of the National Institutes of Health under Award Number U01AG052564 and by funds provided by the McDonnell Center for Systems Neuroscience at Washington University in St. Louis. The HCP-Aging 2.0 Release data used in this report came from DOI: 10.15154/1520707.

## Abbreviations

AD: Alzheimer’s Disease
MRI: Magnetic Resonance Imaging
PRS: Polygenic Risk Score
RBG: Recall-By-Genotype
MCI: Mild Cognitive Impairment
HCP-A: Human Connectome Project– Aging
GWAS: Genome-Wide Association Study
SNP: Single Nucleotide Polymorphism
