# Supplementary material for: Reductions in brainstem volume as a key macrostructural indicator in at-risk populations for Alzheimer’s disease
Source: Alzheimers Res Ther. 2025 Jul 26;17:177. doi: 10.1186/s13195-025-01829-0 (PMC12296611; doi:10.1186/s13195-025-01829-0)
Supplement: Supplementary file 1 — Supplementary Material 1 [file 13195_2025_1829_MOESM1_ESM.docx]

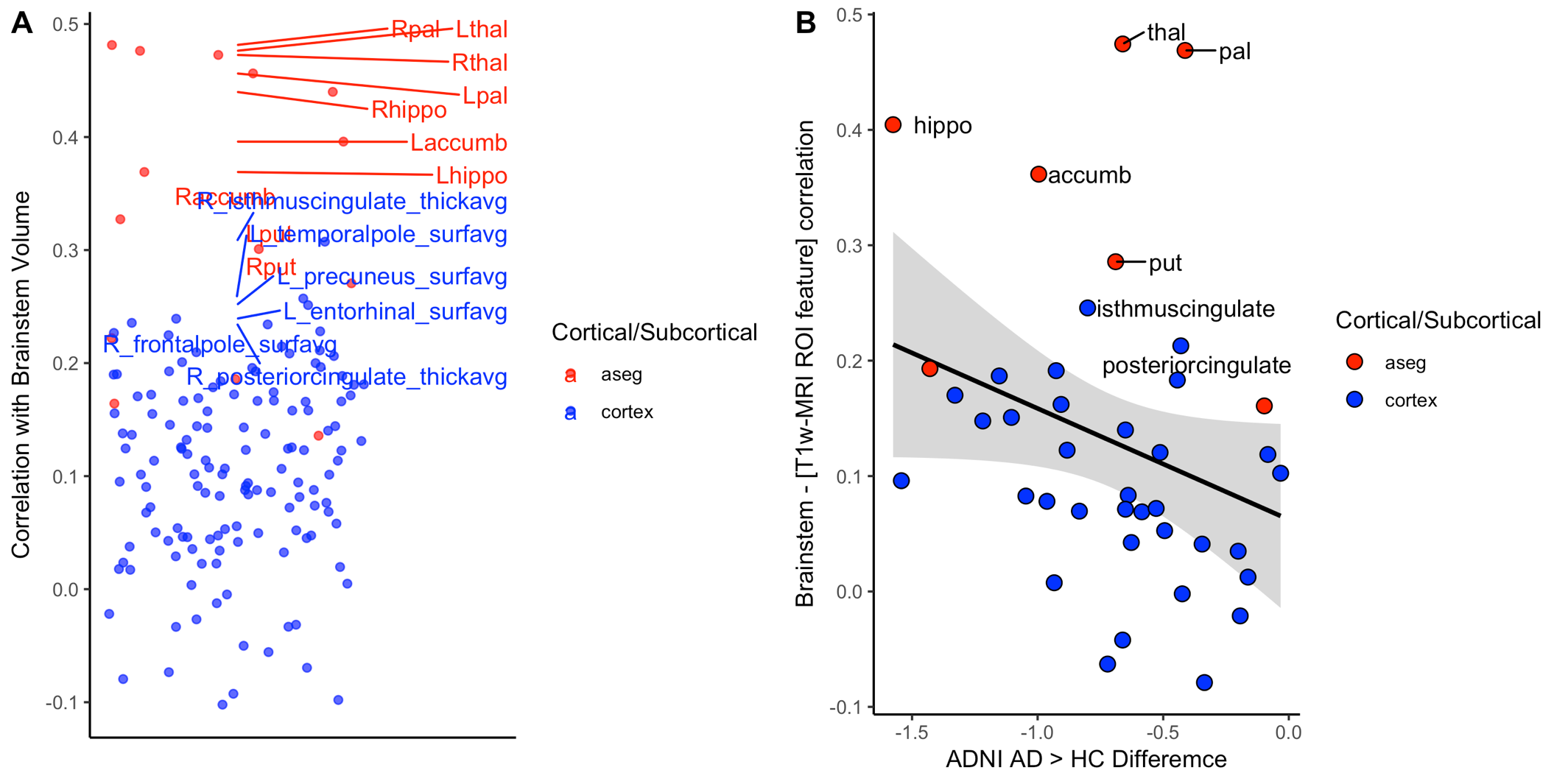


Supplementary Results 1. A) Correlations between brainstem volume and all 150 T1w MRI features, controlling for age, sex and intracranial volume in the HCP-Aging sample. T1w MRI features labelled represent those P _BONFERONNI_ < 0.05 for visualisation purposes. B) Subcortical volumes and cortical thickness correlations were averaged across hemisphere and compared to estimates of Alzheimer’s disease vs healthy aging controls derived from T1w MRI features in the Alzheimer’s disease Neuroimaging Initiative (ADNI) sample, as supplied in ‘RVI’ package in the R environment and previously estimated (1).

1. Kochunov P, Ryan MC, Yang Q, Hatch KS, Zhu A, Thomopoulos SI, et al. Comparison of regional brain deficit patterns in common psychiatric and neurological disorders as revealed by big data. NeuroImage Clinical. 2021;29:102574.
